# Continuation of Report of the Medical Clinic in the Atlanta Medical College, with Some General Views of the Pathology and Treatment of Typhoid Fever

**Published:** 1855-12

**Authors:** J. G. Westmoreland

**Affiliations:** Atlanta, Ga.


ARTICLE III.—*Continuation of Report of the Medical Clinic in the Atlanta Medical College, with some general views of the Pathology and Treatment of Typhoid Fever.* By J. G. WESTMORELAND, M. D., Atlanta, Ga.

During the present year, Typhoid fever being of rare occurrence in the city, it was with some difficulty that suitable subjects of this heretofore very common disease could be often presented to the class in the regular clinical lecture hours. It was the more desirable to do so, on account of the very different views entertained by members ot the medical profession in regard to the etiology, pathology, and treatment of this fever, which, of course, so far from establishing in the mind of the student a settled system of practice, has a tendency to embarrass and confuse the learner.

It is not the large number ot cases, casually noticed by the student, from which benefit is derived, but the presentation by the lecturer of important practical facts connected with its pathology and treatment.

In the pathology, and of course the treatment, laid down by writers on this affection, they differ so materially from each other that the inexperienced are left to form their own opinions of its nature, and the proper management at the bedside of the sick. Clinical lectures will in some degree prepare them to investigate and prescribe readily, provided their attention has been directed to the proper facts and ruling symptoms in the disease.

If he should see but few cases, let him have pointed out the symptoms peculiar to this disease—those which designate it from other fevers; let those symptoms be explained and accounted for in his presence, and he will be better prepared to go forth and assume the responsibility of its management than if he should see a large number of cases which are merely pointed out to him as typhoid fever.

A case brought before the class during the course, not only exhibited the general features of the disease, but went very far to destroy the once somewhat prevalent opinion that typhoid fever rarely if ever occurs in a subject under ten years old. This case was a colored boy, aged four or five years, and before

I saw him had received the usual treatment for remittent fever. In addition to the usual symptoms, extreme irritability of the stomach attended the case when it came under my notice. So constant was this, that for several days almost every article of diet or medicine was rejected in about fifteen minutes after being swallowed. This deprived the patient of the benefit of supporting medicine and food, so necessary to sustain the strength of the system in this protracted disease. From the extreme debility induced by this circumstance it became necessary occasionally to repeat the wine or whatever had been given, as soon as the former portion was rejected. By this process enough was retained to support the declining powers of nature.

In addition to the usual blistering on the nape of the neck, counter irritants to the epigastrium became necessary to moderate the excessive irritability. So prominent was this symptom that a casual examination might have led to the conclusion that the primary derangement was located in the stomach. Hence the importance of determining, from the history and symptoms of the case, the true nature of the disease—whether it be fever or some local inflammatory affection primarily. If it be fever, the first inquiry should be whether there are symptoms known by which the various forms of fever may be determined one from another. If so, what are those symptoms, and to what pathological disturbance do they point? Diagnosis is of the first consideration in the management of a case, the study of which is too often neglected by the physician.

There are many symptoms laid down by writers as evidence of the existence of Typhoid fever; few, however, are peculiar to it. Red tongue, tympanitis, frequent pulse, and tenderness and gurgling sound on pressure in the right illiac region, are said to be the usual symptoms; all of which may be found in other fevers, and may all be absent in some cases of this. It is true that one experienced in the treatment of fevers may, after the disease has progressed several days, form pretty correct conclusions in regard to its type from the general appearance of the patient, and from the tendency and obstinacy of the attack; but to be warranted in fixing the character of the disease during the first few days of its existence, requires most positive and uniformly occurring signs.

Most of the symptoms just enumerated indicate enteritic disturbance, which, unfortunately for the patient, and for correct pathology, very often exist during the progress of the disease in question. As above remarked, these symptoms may be absent. The same may be said of most of the usual symptoms, and still the disease may, with a great deal of certainty, be pronounced Typhoid fever. They are symptoms of secondary disturbance, and can only exist after the disease has progressed sufficiently to induce them.

Many diseases producing a *fever* originate in inflammation of the mucous, serous, glandular and muscular tissues of the body, whose nature is pretty nearly the same as that derangement produced by mechanical injuries to those parts, and take names which express their particular pathological conditions, as pleuritis, peritonitis, &c., &c. Not so with those diseases to which the generic term *fever* has been applied ; for, although local inflammations, in these very tissues, may arise during the progress of fever, our inquiries must extend beyond their existence to arrive at a knowledge of the primary pathological disturbance.

Thanks to the progress of pathological investigation, almost unerring symptoms are known to the profession by which, not only fevers in general are distinguished from local inflammatory diseases, but by which the different varieties of fever may be distinguished from each other ; and that, too, without waiting for accidental developments in their progress.

To the nervous system we look for these characteristics. Not alone to the varying and uncertain symptoms of functional derangement of these organs, but to those unmistakable physical evidences of local derangement of the centres—the cerebrospinal axis.

As the morbific agents, which produce cholera and dysentery, act respectively and specifically upon different portions of the alimentary canal, so also Remittent and Typhoid fevers affect, by the same specific influence, different portions of the spinal cord ; and a train of secondary symptoms may be developed in both these classes of disease.

In the former, the nervous system may exhibit, during their progress, prominent symptoms of disease, and in the latter enteritic symptoms not unfrequently occur ; and notwithstanding these secondary symptoms may become even more prominent than those of the radical lesion or disturbance, and should require special attention in the treatment, yet the primary difficulty should, by all means, be constantly kept in view.

Symptoms of irritation or disease in that part of the spinal marrow corresponding with the first cervical vertebra, extending more or less into the medulla oblongata and down the cervical portion of the cord, manifested by pain, tenderness, &c., are as constantly found in Typhoid fever, as are the evidences of disease in the lower portions of the alimentary canal in dysentery, and require to be regarded in the treatment with equal vigilance.

It has been urged against this theory of local derangement in fevers, that post-mortem examinations reveal no lesion of the nervous centers which will justify the opinion that any impression made on these organs primarily; but it must be remembered that very violent diseases of the nervous system exist without altering materially the appearance, so as to be detected after death. It has, however, yet never been proven that the scalpel reveals less evidences of disease in the nervous system in fevers, than is found after death produced by the ordinary narcotic poisons.

This is a subject worthy of further investigation, and a comparison of the post-mortem appearances from these cases may establish facts in pathology important to the proper understanding of this subject.

The success of the treatment adopted from these pathological views goes far to sustain the correctness of the theory. Alcoholic stimulants, whose action is known to be neutralizing to certain poisonous and depressing influences on the nervous system, and to support the waning energies of the system under such oppression, are important remedies in the treatment of Typhoid fever. The effects of opium and the effects of counter irritants and local depletion on the nuchæ in calming the excitement and irritability of the nervous system, and lessening the violence of the fever, are also evidences of its truth.

Under the deranged state of the nervous energies various tissues are subject to hyperemia, anemia, ulceration, &c., and the general treatment of the fever requires that attention be given to these secondary anormal conditions.

